# Scission-Enhanced Molecular Imaging (SEMI)

**DOI:** 10.1021/acs.bioconjchem.4c00337

**Published:** 2024-09-10

**Authors:** Jeremy M. Quintana, Jonathan C. T. Carlson, Ella Scott, Thomas S. C. Ng, Miles A. Miller, Ralph Weissleder

**Affiliations:** † Center for Systems Biology, Massachusetts General Hospital, 185 Cambridge Street, CPZN 5206, Boston, Massachusetts 02114, United States; ‡ Cancer Center, Massachusetts General Hospital, Boston, Massachusetts 02114, United States; § Department of Radiology, Massachusetts General Hospital, Boston, Massachusetts 02114, United States; ∥ Department of Systems Biology, Harvard Medical School, 200 Longwood Avenue, Boston, Massachusetts 02115, United States

## Abstract

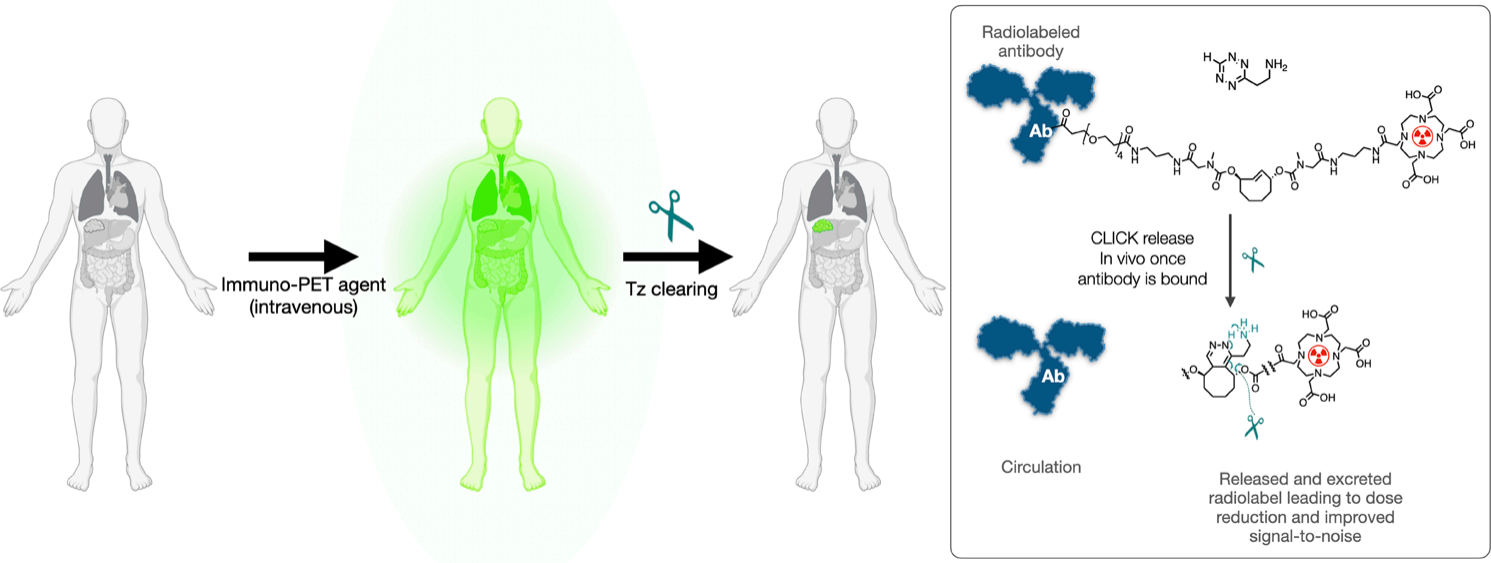

Positron emission tomography (PET) imaging methods have advanced our understanding of human biology, while targeted radiotherapeutic drug treatments are now routinely used clinically. The field is expected to grow considerably based on an expanding repertoire of available affinity ligands, radionuclides, conjugation chemistries, and their FDA approvals. With this increasing use, strategies for dose reduction have become of high interest to protect patients from unnecessary and off-target toxicity. Here, we describe a simple and powerful method, scission-enhanced molecular imaging (SEMI). The technique allows for rapid corporeal elimination of radionuclides once imaging or theranostic treatment is completed and relies on “click-to-release” bioorthogonal linkers.

## Introduction

Positron emission tomography (PET) has enjoyed widespread clinical use following the introduction of hybrid imaging (PET-CT, PET-MRI) and a plethora of ^18^F-labeled small molecule imaging probes over the last three decades.^1^ More recently, immunoPET has developed due to the increasingly rapid approval of therapeutic antibodies and increased production of longer half-life radionuclides (^64^Cu, ^89^Zr, ^68^Ga).^2,3^ Similarly, therapeutic applications of beta and alpha emitters (e.g., ^90^Y, ^177^Lu, ^225^Ac) have rapidly expanded^4^ with the demonstration of clinical efficacy in several oncologic contexts and ensuing FDA approvals.

Several different radionuclide-antibody conjugation strategies have been explored, both for the assembly of the conjugates and functional modulation.^5,6^ Bioorthogonal strategies, in particular,^6^ have received increasing attention since the original description since the methods are fast and orthogonal.^9,10^ The different methods vary in reaction speed and the necessity for further purification.^11^ Most antibody-based radionuclides have long circulation times, and dosing concerns limit the number of scans that can be completed in a patient per year. Similarly, prolonged circulation and off target effects of most therapeutic radioisotopes can cause significant toxicity, requiring suboptimal dosing and/or companion approaches to protect native organs, most notably with renal protecting agents.^12,13^

One way to reduce unnecessary radiation and improve target-to-background ratios is by pretargeting,^14^ which decouples the delivery of the affinity antibody conjugate and radioligand. This approach is often complex and has met varied success in clinical translation. More recently, click chemistries have been used to release payloads from antibodies.^15,16^ Some of these scission-enhanced methods are extraordinarily efficient and enable multiplexed imaging in living tissues^17^ and in vivo.^18^ Here we reasoned that the C_2_TCO-tetrazine click-to-release method^19,20^ could be employed as a clearance strategy to enable dose reductions from unnecessary radiolabels: (i) before imaging, enhancing signal to background, and/or (ii) immediately thereafter, limiting the duration of exposure. We show that in vivo clearance of an immuno-PET agent from circulation can be achieved in a shorter clinically practical time frame and this has potential clinical applications in increasing target signal-to-noise ratios or limiting unwarranted radiation. Additionally, we present a computational model to guide further optimization of this and future immolative PET scaffolds.

## Results

### Design and Synthesis

**Figure 1:**
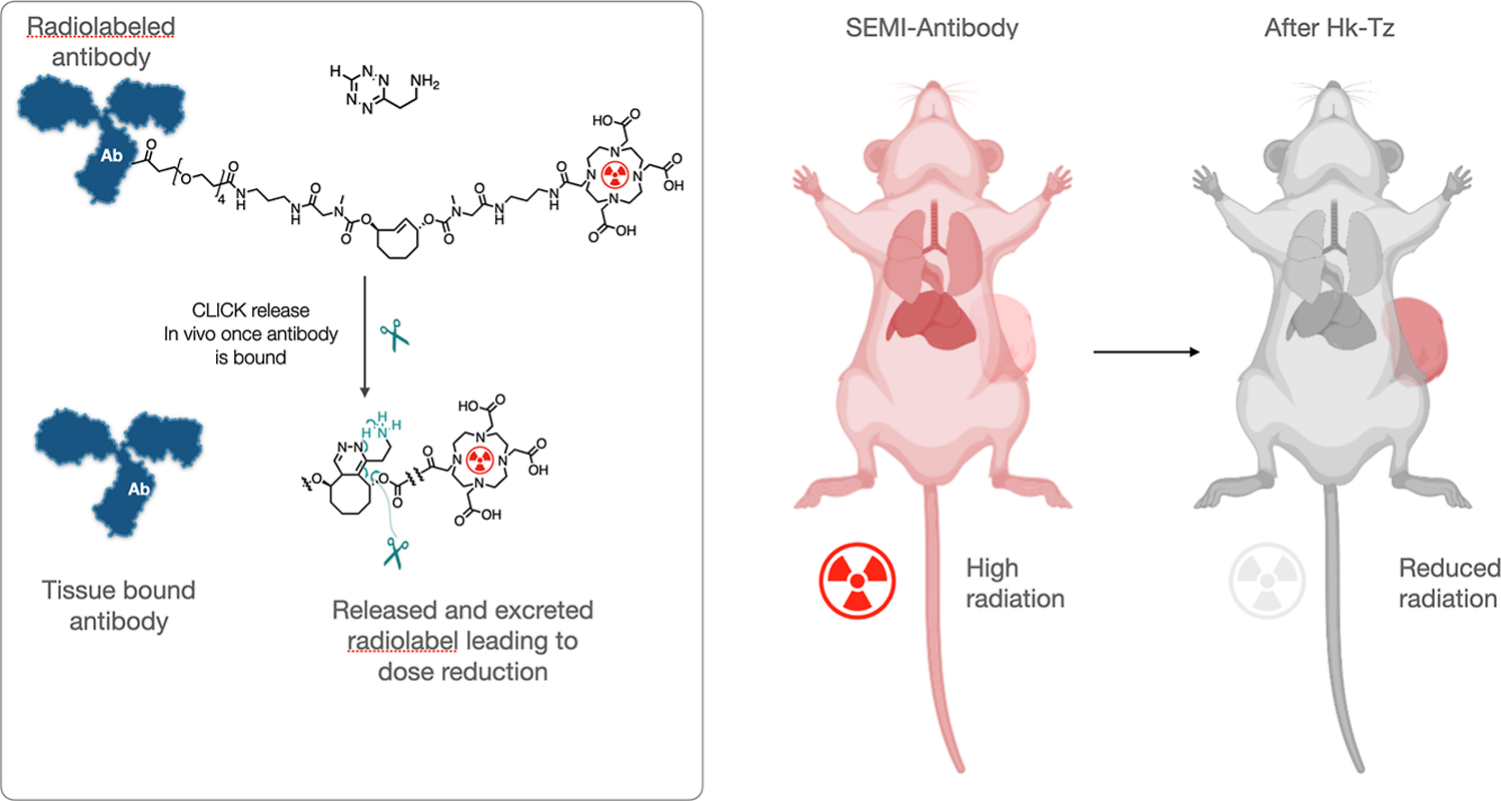
Concept of SEMI. Affinity labels (antibodies, nanobodies, aptamers, proteins, peptides, oligo, nanoparticles) are labeled with a SEMI linker incorporating an immolative TCO moiety such as C_2_TCO. This stable construct is used for in vivo imaging and therapy. When a dose reduction is desired (long circulation time of affinity ligand), a second IV injection of Tz is performed which cleaves the radiolabel-chelator complex in seconds. The latter is rapidly cleared by the kidneys, resulting in dose reduction and favorable imaging/therapy kinetics. Right: example of systemic radiation reduction and on-target specificity increase through SEMI. This strategy is conceptually comparable to bioorthogonal pretargeting, as seen in Figure S1, though SEMI has greater potential to be used with rapidly internalized PET agents.

Figure 1 provides an overview of the probe design. In essence, antibodies are labeled with a SEMI linker incorporating an immolating TCO, such as C_2_TCO. This construct is stable, but it degrades rapidly in the presence of exogenously added tetrazine. The exogenous administration of tetrazine can be custom-selected whenever a dose reduction is desired. Overall, this results in rapid excretion of the radioligand, which can lower the overall radiation dose and potentially increase the signal-to-noise ratio of targeted tumors.

**Figure 2:**
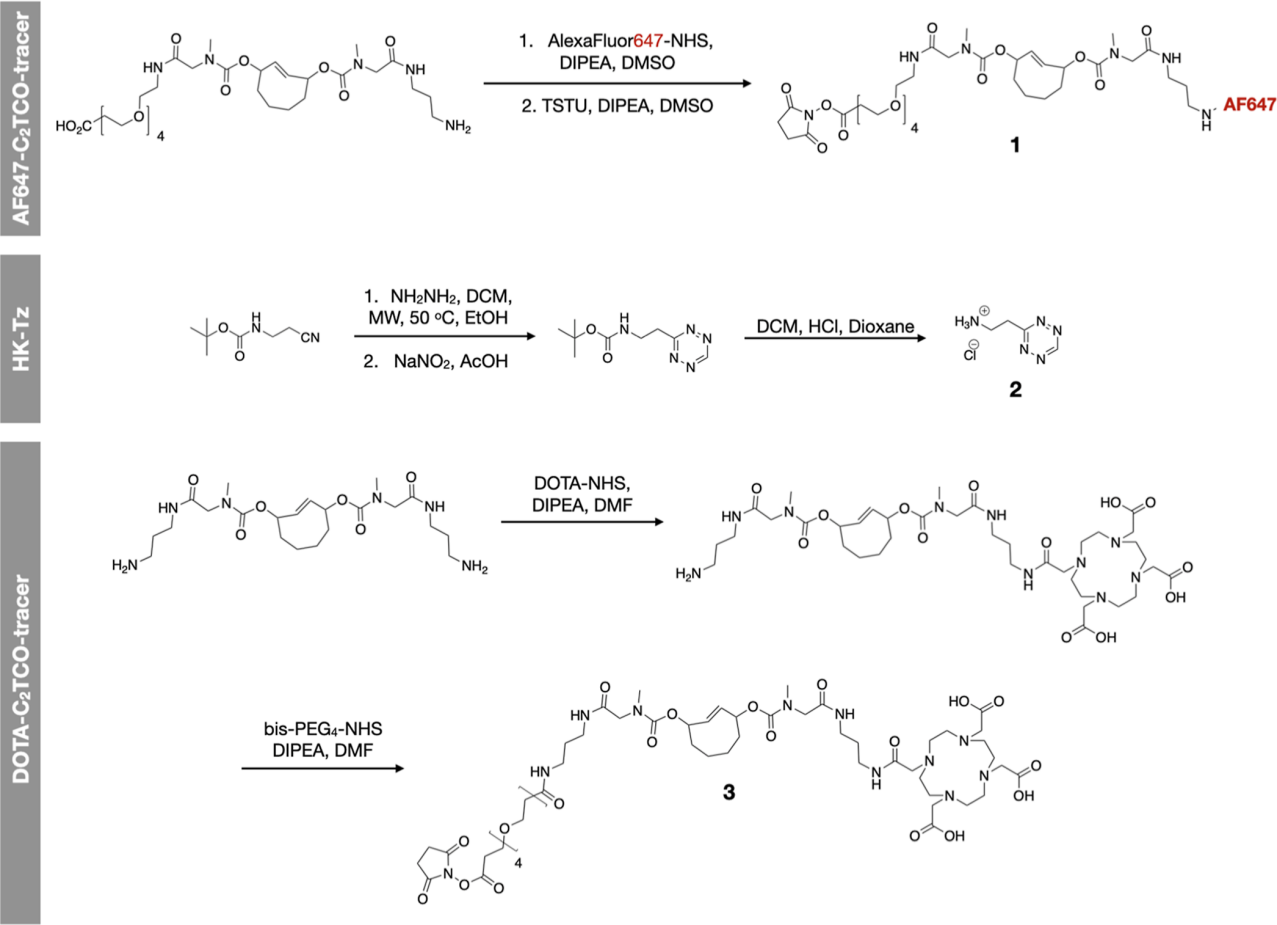
Synthesis of C_2_TCO probes and HK-Tz. SAFE647 was synthesized as described previously^17^ via NHS amide coupling followed by activation of the free carboxylate using TSTU. The resulting NHS-ester was validated via LC–MS and used without further purification. HK-Tz was prepared as previously described to yield the HCl salt.^19^ SEMI tracer was prepared from a symmetrical bis-amino-C_2_TCO,^19^ which was reacted sequentially with NHS-DOTA and bis-PEG4-NHS under basic conditions to provide the desired compound.

We first synthesized the C_2_TCO probes and HK-Tz (Figure 2). The SEMI linker was prepared from a symmetrical bis-amino-C_2_TCO,^19^ which was reacted sequentially with NHS-DOTA and bis-PEG4-NHS under basic conditions to provide the desired compound in a one-pot synthesis. Briefly, the bis-amino-C_2_TCO was dissolved in anhydrous DMF and reacted with 0.8 eq NHS-DOTA in the presence of 1.4 eq DIPEA. This mixture was shaken at room temperature for 1 h before 2 eq bis-PEG4-NHS and a further 1.4 eq of DIPEA were added and the mixture shaken for a further hour. The crude mixture was purified to provide the desired product as a colorless semisolid (59% yield) at >95% purity, as determined by NMR and LC–MS spectrometry (Figures S4–S10). HK-Tz (**2**) was selected as the scission agent for this work due to previous demonstrations of rapid reaction kinetics enabled by the presence of the free amine, which promotes a favorable tautomerization of the initial click product.^19^

### In Vivo Kinetics of SEMI-mAb

**Figure 3:**
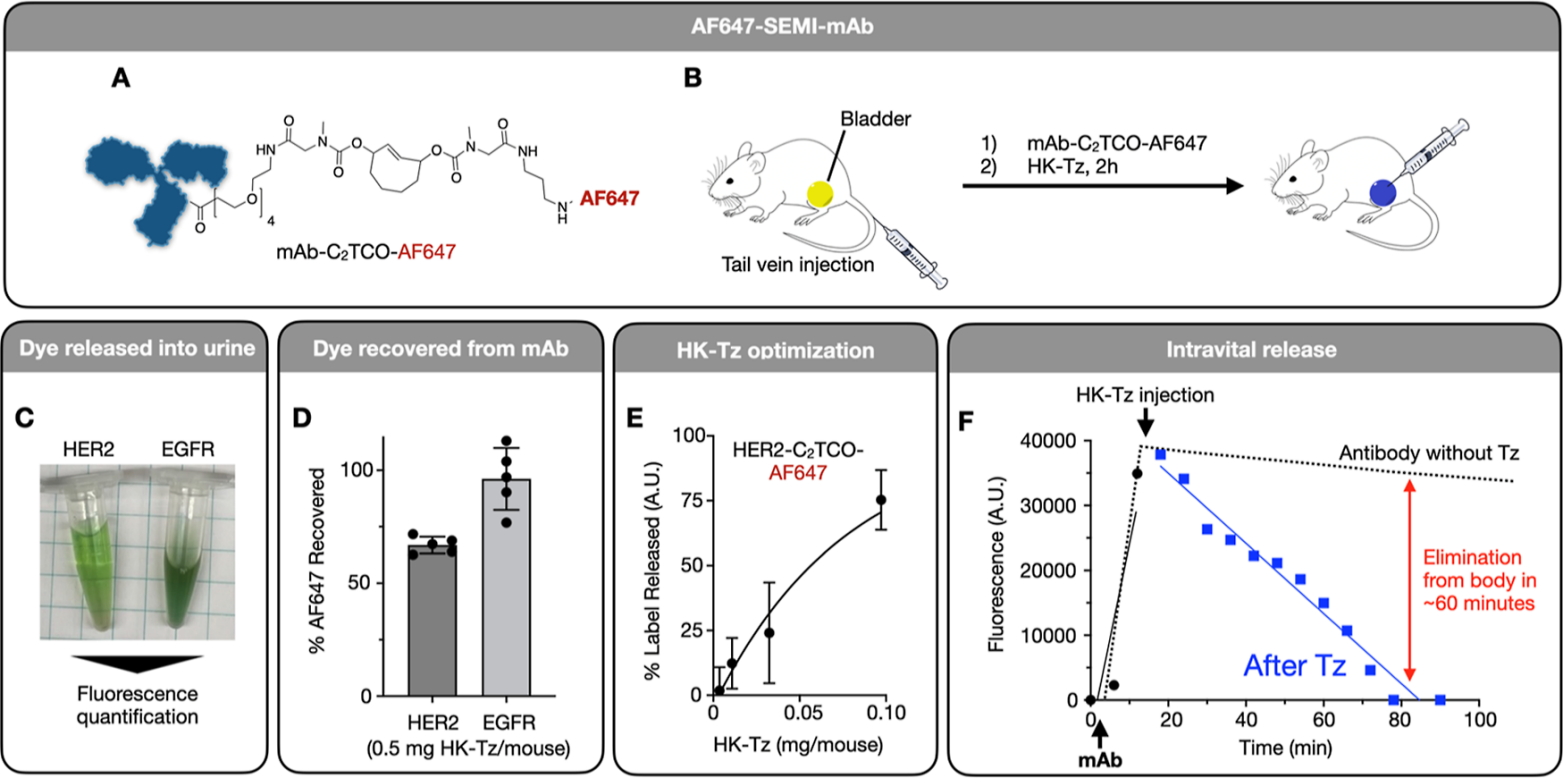
Proof-of-principle in vivo experiments. To determine the kinetics and release of the linker, we used fluorescently labeled HER2 and EGFR constructs and then subjected them to Tz treatment. (A) AF647-C_2_TCO was synthesized as described in Figure 2 and conjugated to antibodies. (B) AF647SEMI-mAb was injected via the tail vein, followed later by tail vein injection of the HK-Tz. 2 h later, the mice were sacrificed, and the urine was collected directly from the bladder. Collected urine was found to be green (C) and was successfully extracted from mice injected with SEMI-cetuximab (EGFR) and SEMI-trastuzumab (HER2); (D) percentage of dye recovered from the bladders of nu/nu mice treated with SEMI antibody after HK-Tz administration (*n* = 2 mice, measurements made in triplicate). (E) After intravenous injection of AF647SEMI-HER2, the ear vasculature was monitored via fluorescence microscopy, and the relative fluorescence within a region of interest was quantified. HK-Tz was injected intravenously after 15 min, and the fluorescence was observed to return to initial levels within 60 min. Fluorescence images were collected every 6 min. (F) Relative fluorescence in mice treated with AF647SEMI-HER2, followed by varying quantities of HK-Tz to determine the optimal dose for in vivo clearance. Measurements were collected in triplicate and reported as means ± s.e.m.

**Figure 4:**
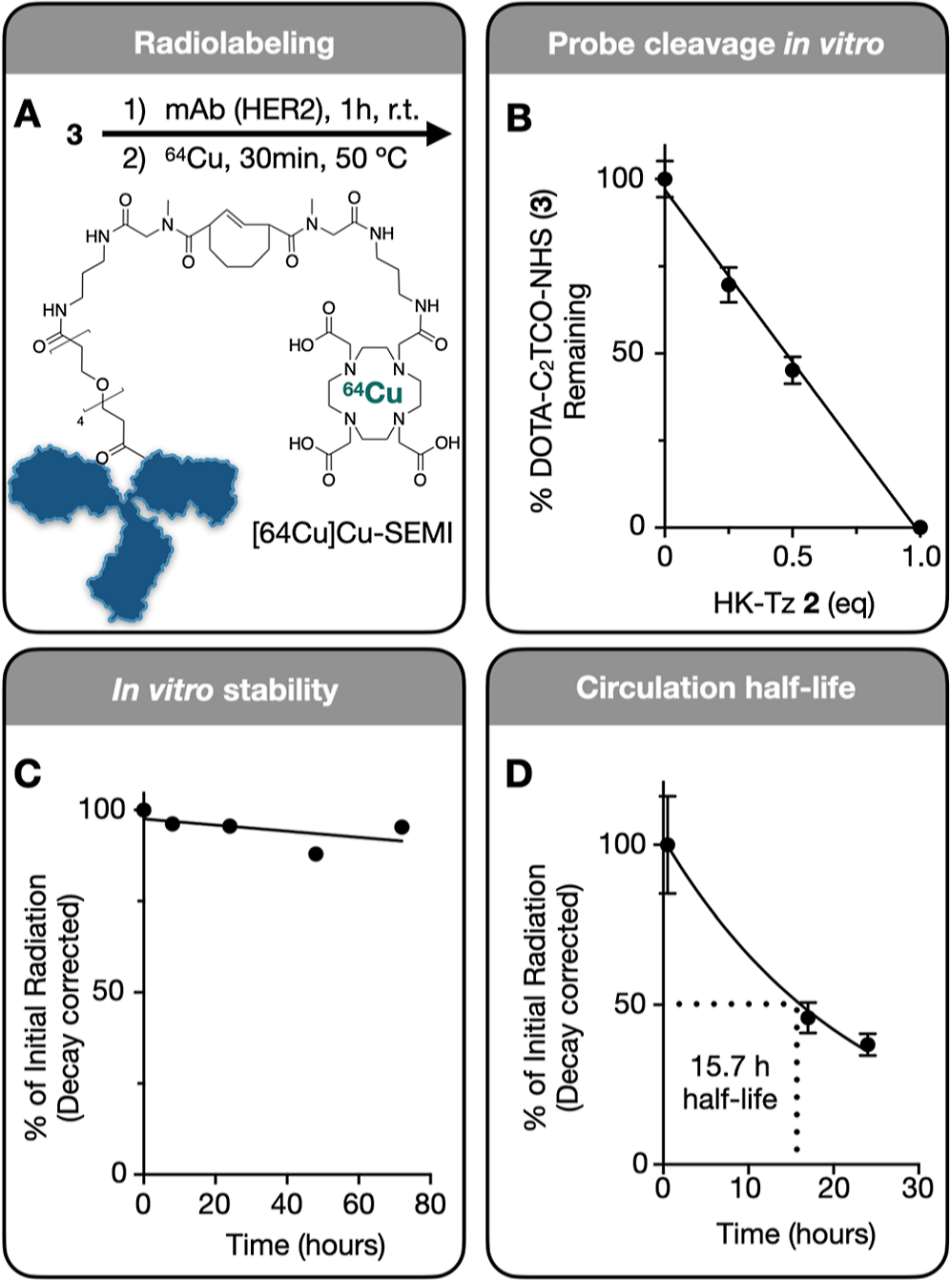
Kinetics of immolative linker. (A) DOTA-C_2_TCO-NHS was synthesized as described in Figure 2, characterized by NMR and conjugated to trastuzumab (anti-HER2). (B) LCMS analysis of DOTA-C_2_TCO-NHS with varying ratios of HK-Tz added in PBS (pH 7.4). (C) Stability of Trastuzumab-C_2_TCO-DOTA loaded with ^64^Cu ([64Cu]Cu-SEMI) over 72 h at 37 °C in 10% fetal bovine serum (in PBS). (D) [64Cu]Cu-SEMI was injected into nontumor-bearing mice, and blood was collected retroorbitally over 24 h. Radiation in the plasma was quantified via gamma counting (corrected for radionuclide decay) to determine the circulation half-life of the SEMI probe in vivo. Data are *n* = 3, means ± s.e.m.

To determine the vascular half-life of labeled antibodies, we first utilized a fluorescently labeled version for microvascular imaging (Figure 3). AF647-C_2_TCO-NHS was conjugated to two antibodies which are used for FDA-approved antibody therapies, trastuzumab (anti-HER2) and cetuximab (anti-EGFR) for proof-of-concept of TCO release in circulation (Figure 3a,b). Mice were treated via tail vein injection of each conjugate (1 nmol of dye) and subsequently with HK-Tz (4 μmol per mouse) after 1 h. Renal excretion of the cleaved fluorochrome fragment yielded green urine, reflecting cleavage of the blue-colored dye (Figure 3c). Analysis of urine fluorescence showed that 60–100% of administered fluorochrome was recoverable (Figure 3d). Optimization of the in vivo SEMI release demonstrated that fully cleaved fluorochrome could be achieved with a dose of 0.1 mg (800 nmol) HK-Tz per mouse (Figure 3e). Additionally, ear vasculature was imaged serially as the AF647SEMI-trastuzumab was administered, and the relative fluorescence intensity was mapped. The data shows a long vascular half-life (∼16 h; Figure 4d) of the antibody as expected unless HK-Tz is administered, upon which the elimination time decreases to ∼60 min (Figure 3f). These data show that SEMI-labeled antibodies retain a long vascular circulation time and can be reduced to background on demand within ∼1 h.

### Kinetics of Immolating Linker

To better understand the kinetics of the immolating DOTA-C_2_TCO-NHS linker, we performed a series of experiments. First, we prepared a trastuzumab-DOTA conjugate using DOTA-C_2_TCO-NHS (Figure 4a), which was then used to chelate ^64^Cu ([64Cu]Cu-SEMI). A solution of the free linker was treated with varying ratios of HK-Tz in PBS and analyzed by LC–MS to verify linker degradation (Figure 4b), and the linker was found to react with the Tz quantitatively. The stability of [64Cu]Cu-SEMI was determined by incubating the conjugate over 72 h at 37 °C (Figure 4c). This data showed that the antibody is stable over extended periods of time without leakage of the linker.

We next determined the amount and timing of HK-Tz required for linker immolation. The mAb conjugate was then injected into nontumor-bearing mice, and blood samples were collected at various time intervals up to 24 h. These samples were centrifuged to isolate the serum, which was analyzed via gamma counting to determine the amount of ^64^Cu present in each sample. The decay-corrected half-life of the antibody was determined to be 15.7 h (Figure 4d). In summary, this data shows that SEMI linker is stable under physiological conditions and demonstrates full *in vitro* release of the chelator in the presence of HK-Tz while maintaining the circulation half-life expected of an antibody conjugate.

### PET Imaging

**Figure 5:**
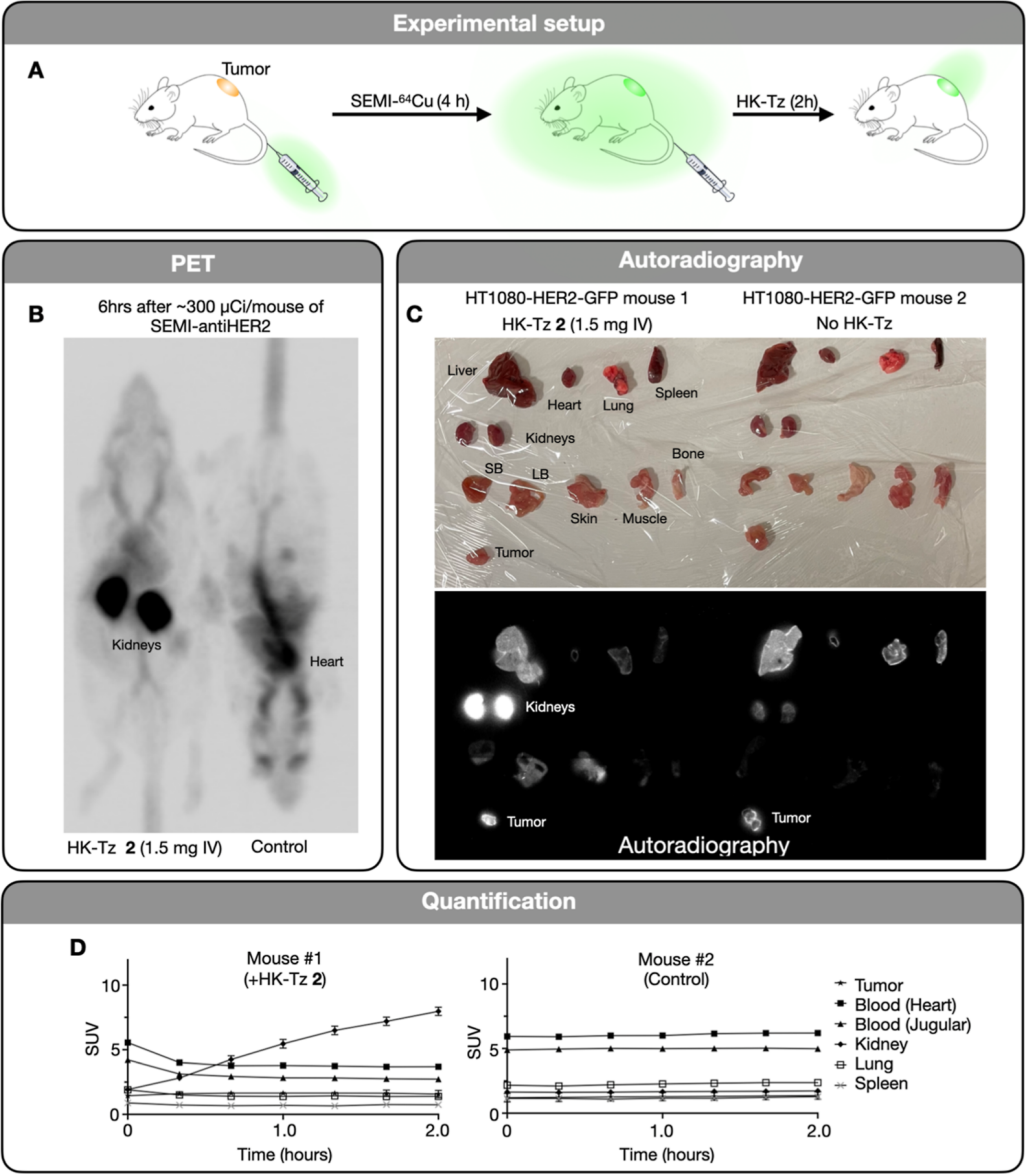
Example of SEMI tracer distribution and PET imaging. (A) Schematic of [64Cu]Cu-SEMI and HK-Tz administration in mice bearing HT1080 (±HER2) tumors. (B) Two mice were injected with [64Cu]Cu-SEMI anti-HER2 antibody. The mouse on the left was given HK-Tz before sacrifice, whereas the mouse on the right was not. After IV injection of HK-Tz, the radioactivity is rapidly cleared, leading to rapid renal clearance. The mouse on the left received HK-Tz, whereas the one on the right did not. Note the higher activity in circulation, liver and rest of the body in the non-Hk-Tz animal. HK-Tz injection leads to lower background and dose reduction (see Figure 7 for modeling of effects). (C) After PET-CT imaging, tissues were harvested, weighed, analyzed via gamma counting, and incubated on a phosphor imaging for 48 h. The phosphor imaging plate was imaged on an Azure Sapphire Biomolecular Imager. (D) SUV measurements of relevant tissues determined from PET imaging.

We next proceeded to PET imaging of SEMI-labeled antibodies. We chose trastuzumab as the model system given its recent clinical development for PET.^21−23^ Additionally, trastuzumab has been shown to be rapidly internalized,^24^ which allows for early scission of the blood pool radiation without significantly affecting the tumor signal intensity. As a model system, we imaged subcutaneous HT1080 xenograft tumors that transgenically overexpress HER2.^25^Figure 5 shows representative PET-CT images of tumor-bearing mice before and after circulating radiation clearance. Mice bearing HER2+ HT1080 tumors were imaged at 4 h after IV administration of the antibody at a time when tumoral accumulation had not yet peaked.^26^ One of the two mice shown in Figure 5b then received an IV injection of HK-Tz, and repeat imaging was performed over 2 h via dynamic PET-CT. Following injection of Tz, the radioactivity is rapidly cleared from the circulation (34–43%) and excreted renally, as quantified by SUV measurements shown in Figure 5c. Findings and tissue distribution were corroborated by autoradiography (Figure 5d) and gamma counting of harvested tissues (Figure 5e). Overall, these experiments show that IV-administered antibodies conjugates can be cleaved in vivo, resulting in reduced unnecessary radiation dose to the body.

**Figure 6:**
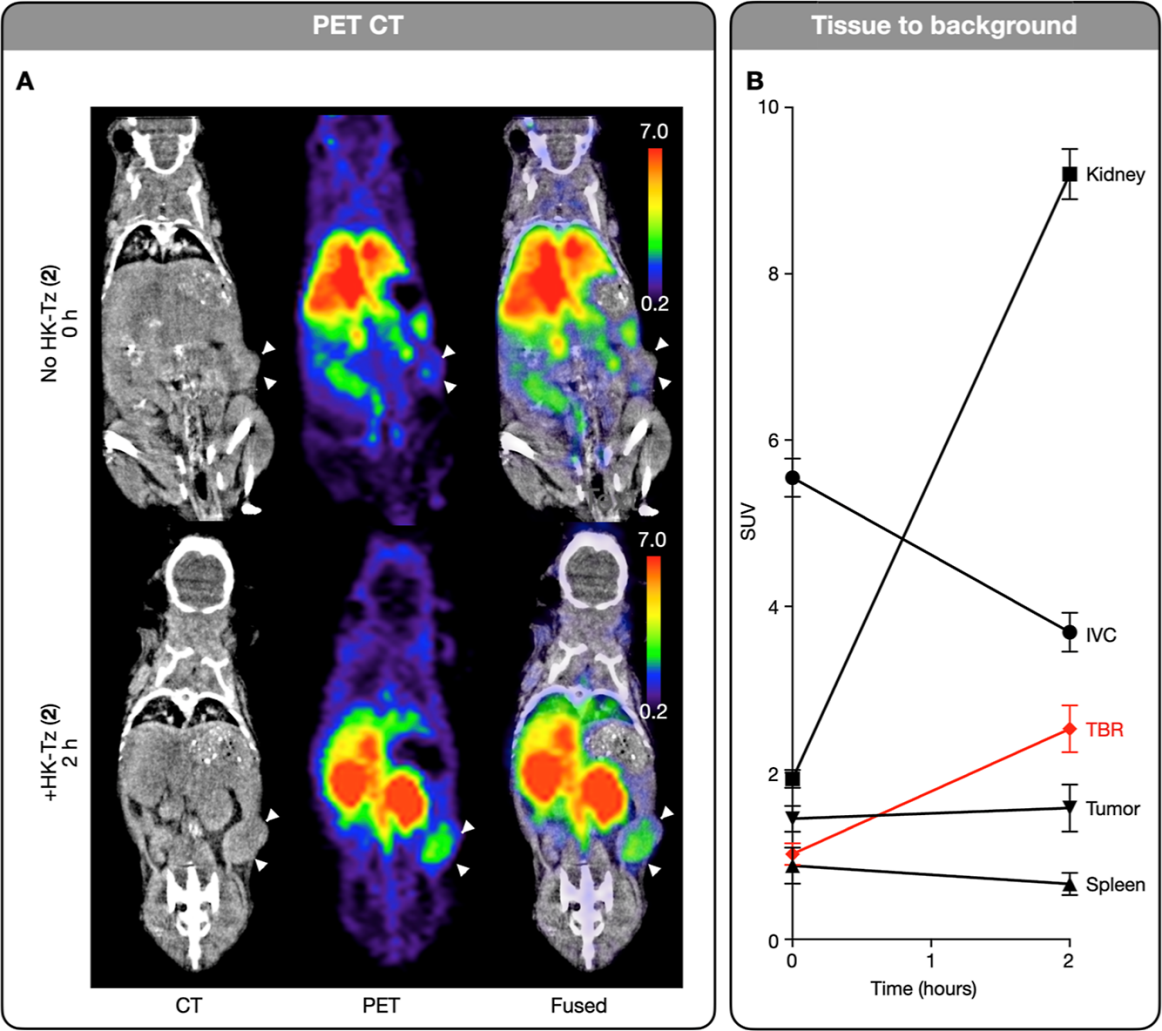
Click-to-release enhances tumor to background. (A) CT and PET images (SUV map) of tumor bearing mice. Tumors are denoted by white arrows. (B) Tissue radiation intensities determined via PET imaging. Tumor to background ratio (TBR) is improved via administration of HK-Tz (**2**) scissors.

PET-CT imaging (Figure 6a) of a tumor bearing mouse before and after HK-Tz treatment reveals that the tumor to background ratio is improved after scission, allowing for improved visualization of the tumor after 2 h. These PET images were analyzed to quantify this ratio (Figure 6b). Tissues were then harvested from the mice, as well as control mice which did not receive HK-Tz, and the radiation from each was determined via gamma counting (Figure S3a). This biodistribution analysis further demonstrates that HK-Tz scission reduces off target radiation, while signal in the tumor is minimally impacted.

### Modeling

**Figure 7:**
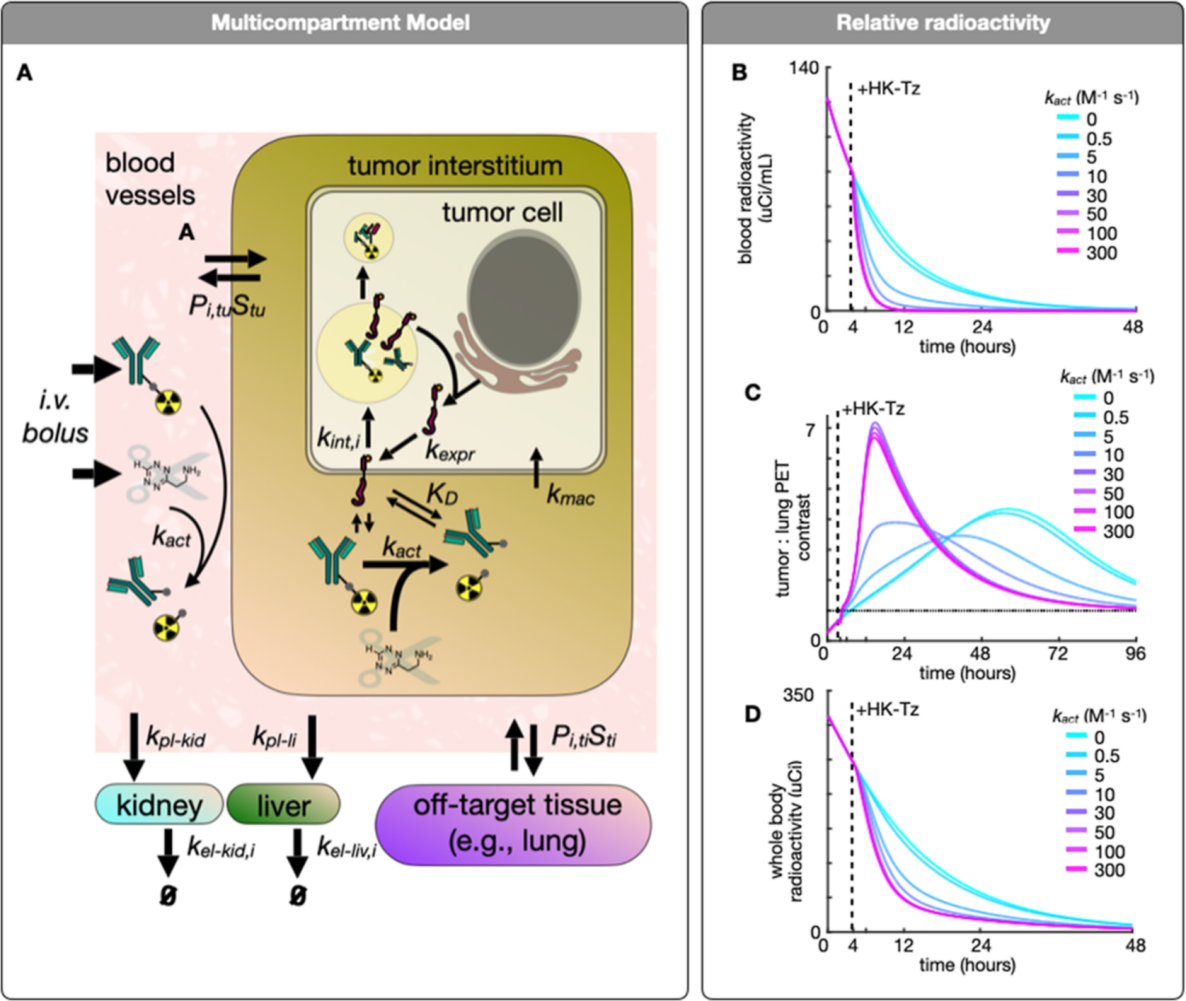
Modeling of SEMI PET imaging. (A) Multicompartment SEMI model to interpret tissue concentrations over time (see Figure S11 for details). (B) Relative blood radioactivity following IV administration of SEMI-antibody. The injection of Hk-Tz is comparable to a *k*_act_ of ∼10 M^–1^ s^–1^ at concentrations used in this study. The “*k*_act_” is the bimolecular rate constant for the click reaction. At 30× higher concentrations, the *k*_act_ can be increased to ∼300 with near complete and immediate clearance of blood activity. (C) Effect of Hk-Tz on tumor/lung imaging ratio at different *k*_act_. (D) Modeling the effects of HK-Tz administration on total body radiation, which may have applications in radiotheranostics.

To help interpret SEMI PET imaging, we developed a simplified computational model to describe the transport and chemical reactions of SEMI components in different tissues in mice. The model uses coupled ordinary differential equations to describe transport from blood into tissues, including the tumor, the lungs as a model off-target tissue, and the liver and kidneys as the primary clearance organs (Figure 7a). The model incorporates antibody binding and cellular internalization, Tz-mediated linker release, reagent transport to and from the blood into tissues via convection/diffusion, and renal/hepatobiliary excretion. We drew rate constants and parameters from prior literature, and a subset of features were fit to the PET imaging data (Table S1). The optimized model accurately matched PET imaging measurements (*R*^2^ = 0.91, Figure S11).

To estimate how SEMI affects the relative signal in tumors compared to off-target tissues, we adjusted the bimolecular rate constant for SEMI linker cleavage (*k*_act_) and simulated the resulting behaviors of the system. The model indicated that efficient SEMI cleavage leads to rapid clearance of radioactivity from the blood (Figure 7b), increases the level of imaging signal in on-target HER2+ tumor tissue compared to off-target tissue (Figure 7c), and accelerates the excretion of radioactive material from the body (Figure 7d). Of note, the effect of an increased *k*_act_ cleavage rate can also be achieved by increasing the administered dose of HK-Tz. Overall, the computational model provided an estimate of rate-constants and parameters for in vivo SEMI action, and further supported how SEMI can be used to improve specificity in tumor-targeting while decreasing unwanted radiation exposure to off-target tissues.

We estimated how SEMI may impact the relative dose imparted to different tissues with and without scission using the simulated time activity curves above (Figure S3b). Notably, the absorbed dose decreased for all tissues except for the kidneys. The elevated renal dose remains well under the threshold expected to cause significant radiation-induced renal injury.^27^

## Discussion

The current research describes an on-demand immolating linker for in vivo radionuclide imaging and therapy. Recent years have seen an increase in the use of antibodies and other biologicals for in vivo use. A key defining factor of these molecules is their long circulation times. While this is desirable in antagonists (blocking a biological function), it is less desirable in radionuclide-based applications as long circulation times add (i) unnecessary radiation to undesired sites and (ii) decrease the signal-to-noise ratio. For example, it is estimated that a single 130 MBq ^64^Cu dose of trastuzumab constitutes a significant amount of the annually allowed radiation dose^28,29^ thus limiting the number of scans that should be done in a patient per year. Given the need for multiple other imaging tests and other procedures requiring ionizing radiation per patient, it is therefore desirable to reduce unnecessary radiation. Using the approach described here, the radionuclide-chelate can be cleaved from the antibody and excreted within a short time frame once imaging is complete. The method thus represents a new approach to dose reduction in nuclear imaging and complements alternative approaches such as pretargeting.^10^

Steady development of click reactions over the last 20 years has led to an expanding gamut of applications,^7^ including a range of radiolabeling techniques, where the tools have become a method of choice since their first description.^8^ Here, we apply click tools not to deliver labels but to remove them via bioorthogonal bond cleavage.^30,31^ Whereas past investigations of bioorthogonal ligation reactions have indicated a need for rate constants >50,000 M^–1^ s^–1^ for efficient performance in vivo,^32^ here, we observe unexpectedly efficient scission despite significantly slower reaction kinetics on the order of 100–400 M^–1^ s^–1^ for the Tz scissors click reaction with C_2_TCO. The strategy chosen allows efficient, irreversible cleavage while using readily achievable concentrations of reagents. The SEMI reagents thus achieve a unique combination of high intrinsic biochemical stability (C_2_TCO-DOTA, >97% intact at 48 h) and facile, high-yielding reactivity in tetrazine-triggered cleavage. We show that the Tz-C_2_TCO cleavage is >95% complete in 60 min using HK-Tz in vivo.

The proof-of-principle experiments shown here could be further refined and expanded for in vivo use. An ideal time frame for administration of the scission agent will need to be optimized for each antibody/target pair, as slower rates of tumor uptake and internalization will require later treatment to avoid undesired reduction of the tumor signal. Likewise, development of novel antibodies to improve rates of internalization would be beneficial to the efficacy of this platform. Other immolative TCO isomers (cTCO,^15,33^ sTCO,^34^ dcTCO^35^) could be utilized in combination with alternative functionalized tetrazines,^33,36−38^ to release any of the multitude of chelators (NOTA, DFO, macropa)^2,39^ currently being utilized for radioligand preparation. A combination of TCO/Tz pairs exhibiting faster second-order kinetics is now possible and could allow for more efficient release with less Tz required, while alternative chelators could be used to expand the radionuclides that can be applied to this platform. Thus, this immolative platform has the potential utility to decrease off-target delivery of chelated therapeutic nuclides. Finally, while our focus here was on demonstrating the proof-of-principle, the new approach opens the door to a broad range of imaging and theranostic use to improve signal-to-noise ratios, reduce unnecessary radiation, and potentially allow multiplexed nuclear imaging.

## Materials and Methods

### Materials

Solvents, reagents, and enzymes were purchased from Sigma-Aldrich (St. Louis, MO) and used without further purification unless otherwise stated. An aqueous solution was prepared using Milli-Q water (Millipore). AlexaFluor647-NHS and DOTA-NHS were obtained from Click Chemistry Tools (Scottsdale, AZ) and Macrocyclics, Inc. (Plano, TX), respectively. ^64^CuCl_2_ was purchased from the Department of Medical Physics in the University of Wisconsin (Madison, WI). Antibody (trastuzumab) was generously provided by Genentech (South San Francisco, CA).

### Probe Synthesis and Conjugation

AF647SEMI-NHS ester (**1**) was synthesized as described previously.^17^ Briefly, HO_2_C-C_2_TCO-NH2 was dissolved in anhydrous DMSO and reacted with AlexaFluor647-NHS ester and DIPEA for 30 min at room temperature. The product was then activated by adding TSTU (1 equiv) and DIPEA (1 equiv). After shaking vigorously for 1 min, this mixture was analyzed via LCMS and determined to match the desired product **1**, which was used without any further purification.

**MS (+ESI)***m*/*z*: calcd for C_71_H_103_N_8_O_27_S_4_^+^[M + H]^+^, 1627.58; found, 1627.97.

DOTA-C_2_TCO-NHS was synthesized by dissolving C_2_TCO-diamine (10 mg, 20.6 μmol) in anhydrous DMF (1 mL) and adding NHS-DOTA (11.4 mg, 15.0 μmol) and DIPEA (5 μL, 28.7 μmol). This mixture was shaken at room temperature for 1 h, then bisNHS-PEG4 (22 mg, 45.0 μmol) and DIPEA (5 μL, 28.7 μmol) were added, and the mixture was shaken for a further hour. The crude mixture was loaded directly onto a reverse-phase column and purified using a gradient of 5–95% acetonitrile in water (0.1% formic acid). Fractions containing the product were identified via LC–MS, then combined and evaporated to provide the product (3) as a colorless semisolid (15.1 mg, 59% yield).

^**1**^**H NMR** (400 MHz, CDCl_3_): δ 12.14 (br s, 3H), 8.13 (s, 1H), 7.95 (s, 1H), 7.82 (2H), 5.78 (t, *J* = 27.9 Hz, 1H), 5.25 (t, *J* = 20.3 Hz, 1H), 3.95–3.87 (m, 1H), 3.83 (t, *J* = 6.8 Hz, 1H), 3.71 (t, *J* = 6.0 Hz, 4H), 3.59 (t, *J* = 6.3 Hz, 6H), 3.55–3.51 (m, 8H), 3.50–3.45 (m, 24H), 3.15–3.00 (m, 6H), 2.92 (t, *J* = 5.82 Hz, 6H), 2.86 (t, *J* = 5.7 Hz, 2H), 2.81 (s, 8H), 2.59 (s, 1H), 2.44 (t, *J* = 6.3 Hz, 4H), 2.29 (t, *J* = 6.5 Hz, 2H), 2.00–1.86 (m, 1H), 1.58–1.48 (m, 4H), 1.10–0.94 (m, 1H).

^**13**^**C NMR** (101 MHz, CDCl_3_): δ 184.3, 172.8, 172.7, 170.2, 168.6, 167.4, 162.3, 155.1, 154.7, 129.4, 73.7, 69.8, 69.7, 69.6, 69.5, 66.9, 66.2, 65.2, 53.3, 41.7, 40.4, 38.3, 36.2, 35.8, 34.8, 31.6, 30.8, 29.3, 25.5, 25.2, 23.3, 17.6, 12.7.

**MS (+ESI)***m*/*z*: calcd for C_54_H_90_N_11_O_22_^+^[M + H]^+^, 1244.63; found, 1244.90.

Conjugation of these probes was performed by preparing trastuzumab or cetuximab in PBS (pH 7.4, 2 mg/mL). To these solutions was added a solution containing eight eq of either AF647SEMI-NHS (**1**) or SEMI (**3**) in dry DMF (2 mM) and saturated sodium bicarbonate to achieve a final pH of ∼8.5. These solutions were shaken at room temperature for 1 h, then any remaining probe was removed by spin filtration (50 kDa MWCO, 7000 rcf for 5 min, ×5 in PBS). The solutions were then diluted to a concentration of ∼1 mg/mL in PBS and stored at 4 °C until further use (final DOL ≈ 3.5, Figure S1b).

### ^64^Cu Labeling of Antibody

Metal free buffer solutions were prepared using Chelex 100 Chelating Resin (100–200 mesh, BioRad). SEMI-trastuzumab (50 μg) was diluted with citrate buffer (400 μL, 0.1 M, pH 5.0). ∼25 mCi (∼925 MBq) of ^64^CuCl_2_ in 0.1 N HCl was mixed with citrate buffer (0.1 M, pH 7.0) to form ^64^Cu(OAc)_2_ and pH was adjusted to ∼5. The antibody was labeled with ^64^Cu at 50 °C for 30 min on a thermomixer (900 rpm). The labeling efficiency was monitored by iTLC showing >99% labeling with a specific activity of 19.2 MBq (514 μCi) ^64^Cu/μg antibody. Trace amounts of unchelated ^64^Cu were removed by EDTA chelation (final concentration ∼5 mM) followed by centrifugation (MWCO 50 kDa) at 10k × g for 5 min. The buffer was exchanged to PBS, and [64Cu]Cu-antibody was sterilized using a 0.22 μm HT Tuffryn membrane string filter (PALL) in ∼167 MBq (∼4.5 mCi) [64Cu]Cu-antibody with ∼95% average decay-corrected radiochemical yield (RCY). iTLC demonstrated >99% radiochemical purity of ^64^Cu-antibody.

### Cell Lines and Animal Models

HT1080 human cancer cells were derived from fibrosarcoma, obtained from ATCC and stably transfected with a HER2-GFP fusion construct as previously described.^25^ All animal research was performed following guidelines from the Institutional Subcommittee on Research Animal Care. HT1080-HER2-GFP tumors were generated by injecting 1 million cells subcutaneously and contra-laterally in the flanks of 7–12 week-old female nu/nu mice (MGB Cox 7 Core). For all procedures, mice were anesthetized with an isoflurane vaporizer on a heated stage; euthanasia was performed by CO_2_ chamber when necessary, and all treatment groups underwent procedures and monitoring consecutively on the same day when possible, but in a randomized order.

### Intravital Imaging

All confocal images were collected using a customized Olympus FV1000 confocal microscope (Olympus America). A 2× (XLFluor, NA 0.14), a 4× (UPlanSApo, NA 0.16), and an XLUMPlanFL N 20× (NA 1.0) water immersion objective were used for imaging (Olympus America). Hoechst nuclear staining, Dextran-AF555 (Thermo), and AF647SEMI-mAb (mAb: Herceptin, Trastuzumab) were excited sequentially using a 405 nm, a 559 nm, and a 633 nm diode laser, respectively, in combination with a DM-405/488/559/635 nm dichroic beam splitter. Emitted light was further separated by beam splitters (SDM-473, SDM-560, and SDM-640) and emission filters BA430-455, BA490-540, BA575–620, and BA655-755 (Olympus America). Confocal laser power settings were carefully optimized to avoid photobleaching, phototoxicity, or damage to the brain. All images were processed using Fiji (ImageJ2, Vers.2.3/1.53f).

Blood half-life (*t*_1/2_) measurement of the labeled antibody was performed using confocal imaging. Nu/nu mice (MGB Cox 7 Core) were anesthetized using isoflurane, stabilized using a stereotaxic (Kopf, Tujunga, CA) for motion-free imaging, and a vascular probe was injected to select areas in the ear with good vasculature for imaging. Time-series of confocal imaging stacks in multiple locations were initiated before injection of fluorescent AF647SEMI-mAb (0.15 mg) via tail vein catheter. After 15 min, HK-Tz (0.1 mg in 100 μL PBS) was injected while Z-stacks from the area were collected every 6 min over 2 h. Average fluorescence signal intensity was quantified using six ROIs, each inside the vasculature and outside of the vasculature (background) using Fiji (ImageJ2, Vers.2.3/1.53f). Background fluorescence was subtracted from the average signal inside the vasculature, and the values were analyzed and plotted in GraphPad Prism (San Diego, CA, Version 9.3.1 for Mac).

### PET Imaging

The PET-CT imaging procedure was similar to previously described methods.^40^ PET-CT imaging was performed roughly 4 h after tail-vein injection of [64Cu]Cu-antibody (9.6 ± 2.4 MBq/230 ± 64.3 μCi in 150 ± 10 μL). High-resolution CT (Inveon, Siemens, Munich, Germany) was conducted prior to the PET scan. The CT scan was acquired with X-ray power of 80 kVp and 500 μA, an exposure time of 370 to 400 ms, and an isotropic resolution of 90 μm. The ordered subsets expectation maximization (OSEM) and filtered back projection (FBP) algorithms were used for PET imaging reconstruction to obtain a spatial resolution approaching approximately 1 mm. For quantitative PET analysis, regions of interest were defined on the basis of anatomic CT data. For autoradiography, tissues were exposed to a storage phosphor screen in a cassette (GE Healthcare) for ∼24 h and visualized using a Azure Sapphire Biomolecular Imager (Azure Biosystems).

### Dosimetry

Absorbed dose across tissues due to 64Cu-SENIT with and without scission was estimated using MIRD formalism.^41^ Tissue time-integrated activity curves (TIAC) with and without cleavage were estimated using curves generated from simulation models presented in Figure 7. *S*-values were estimated from Monte Carlo simulations of a 25 g mouse phantom.^42^ Tumors were inoculated subcutaneously; therefore, the *S*-value derived for skin was used to estimate the tumor *S*-value.

### Statistics

Unless otherwise indicated, results are expressed as mean ± SEM throughout. Statistical analyses were performed using Prism (GraphPad), MATLAB (Mathworks), and Excel (Microsoft). Two-tailed tests, ANOVA tests, and spearman correlation tests were used with false-positive thresholds of α = 0.05.

### Computational Modeling

Simulations were performed in MATLAB 2022b (Mathworks) using the ode15s solver. Parameters used literature values reported in Table S1. Parameter optimization used a global optimization function Gray Wolf Optimizer^43^ with least-squares cost function to fit time-lapse SEMI PET imaging data. Modeled tissue concentrations incorporated estimates of the vessel volume fraction for individual tissues as reported in prior literature.

## Supporting Information Available

Additional experimental and computational details and NMR spectra for novel compounds (PDF)
