# Supplementary material for: Scission-Enhanced Molecular Imaging (SEMI)
Source: Bioconjug Chem. 2024 Sep 10;35(10):1543–52. doi: 10.1021/acs.bioconjchem.4c00337 (PMC11488501; doi:10.1021/acs.bioconjchem.4c00337)
Supplement: Supplementary file 1 — bc4c00337_si_001.pdf [file bc4c00337_si_001.pdf]

Supplemental information

Scission-enhanced nuclear imaging and treatment (SEMI)

Jeremy M. Quintana[1,3], Jonathan C. T. Carlson[1,2]*, Ella Scott[1], Thomas S.C. Ng[1,3], Miles A. Miller[1,3], Ralph Weissleder[1,2,3,4]*

[1] Center for Systems Biology, Massachusetts General Hospital, 185 Cambridge St, CPZN 5206, Boston, MA 02114,

[2] Cancer Center, Massachusetts General Hospital, Boston, MA 02114

[3] Department of Radiology, Massachusetts General Hospital, Boston, MA 02114

[4] Department of Systems Biology, Harvard Medical School, 200 Longwood Ave, Boston, MA 02115

*R. Weissleder, MD, PhD
Center for Systems Biology
Massachusetts General Hospital
185 Cambridge St, CPZN 5206
Boston, MA, 02114
617-726-8226
rweissleder@mgh.harvard.edu (contact)
carlson.jonathan@mgh.harvard.edu

Keywords: PET, antibody, theranostics, radiation, dose reduction, diagnostics, therapy

	Bioorthogonal pretargeting	SEMI
**Scheme**	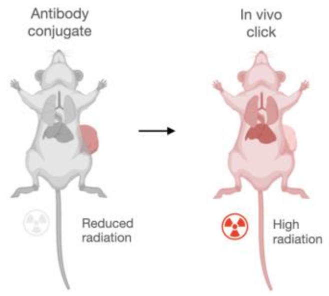	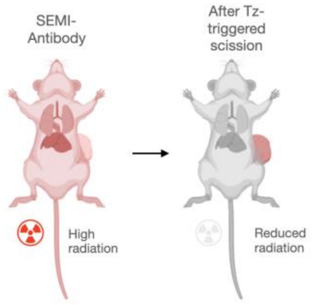
**Reagents**	*In vivo click*<br>TCO target<br>Tz-radiotracer	*Click-to-release*<br>Various TCO linkers<br>Tz-scissors
**Extra steps**	Clearing agents	*None*
**Tissue Targets**	**Non**-internalizing	Internalizing
**Radiocarrier**	Reactive Tz	Stable mAb
**Goals**	Maximize TBR<br>Short radiotracer half-life	Reduce radiation exposure<br>Increase TBR<br>Enable radiopharmaceutical therapy (RPT)

Fig. S1: Comparison of bioorthogonal pretargeting (“in vivo click”) and SEMI (“in vivo click to release”).

A

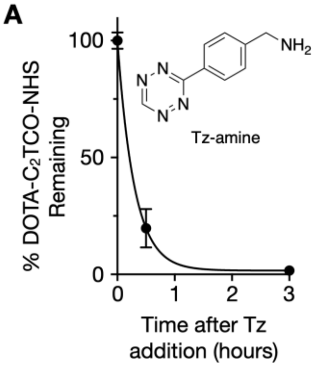

B

	Antibody Concentration (μM)	AF Concentration (μM)	DOL
AF647-SENIT-mAb			
Trastuzumab	4.36	15.22	3.5
Cetuximab	3.88	14.81	3.8
SENIT (no ^{64}Cu)			
Trastuzumab	4.73	9.63	4.1*

Figure S2. Additional kinetics and characterization of SEMI probes. A) Kinetics of SEMI linker release. DOTA-C_2TCO-NHS ester, at a concentration of 100 uM in PBS, was treated with 1 mM of the commercially available Tz-amine, and samples were analyzed by LCMS after 30 minutes and 3 hours. Even when triggered by this non-optimal tetrazine, the C_2TCO linker demonstrated a half-life of ~12 minutes. Data are n = 3, means ±s.e.m. B) Degree of labeling (DOL) of antibody conjugates used in these studies. For AF647 conjugates the concentrations of the antibodies and fluorophore were directly measured from a 100-fold dilution of the stock solution. For the SEMI (no ^{64}Cu) conjugate with DOTA-C_2TCO-NHS linker, an aliquot of the conjugate was first reacted with excess mTz-AF488 for 1 hour in PBS. This click-product was then purified using a Zeba desalting column (Thermo, USA) before quantification. All concentrations were determined using a Nanodrop 1000 to measure the relevant absorbance values (280nm for mAb, 495nm for AF488, and 650nm for AF647), which were then applied to Beer's law. In the case of the 488 conjugate, half of the dye is expected to be eliminated after tetrazine ligation due to the cleavage of the TCO linker, so the DOL was adjusted accordingly.

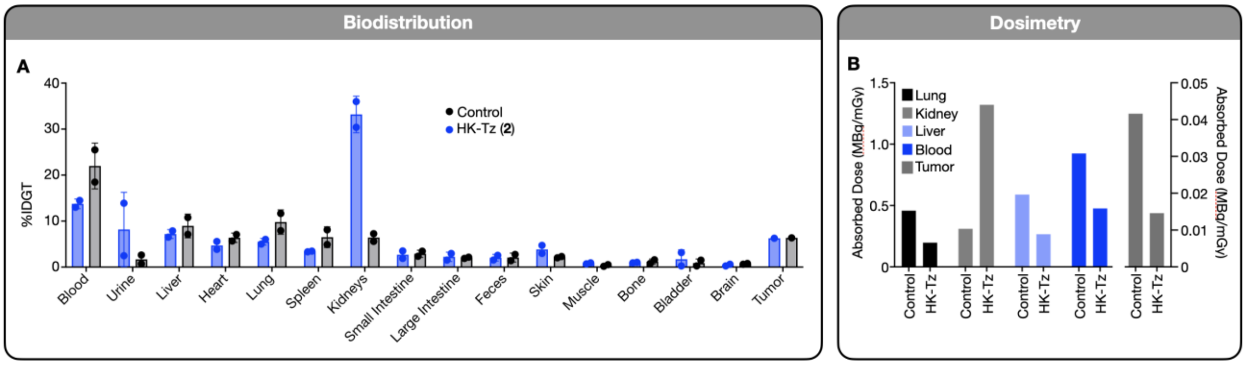

Fig. S3. **A.** Biodistribution of radiation with and without HK-Tz (**2**) scissors. **B.** Dosimetry of selected tissues demonstrating the reduction of absorbed dose. Data is from the mouse depicted in **Fig. 6**.

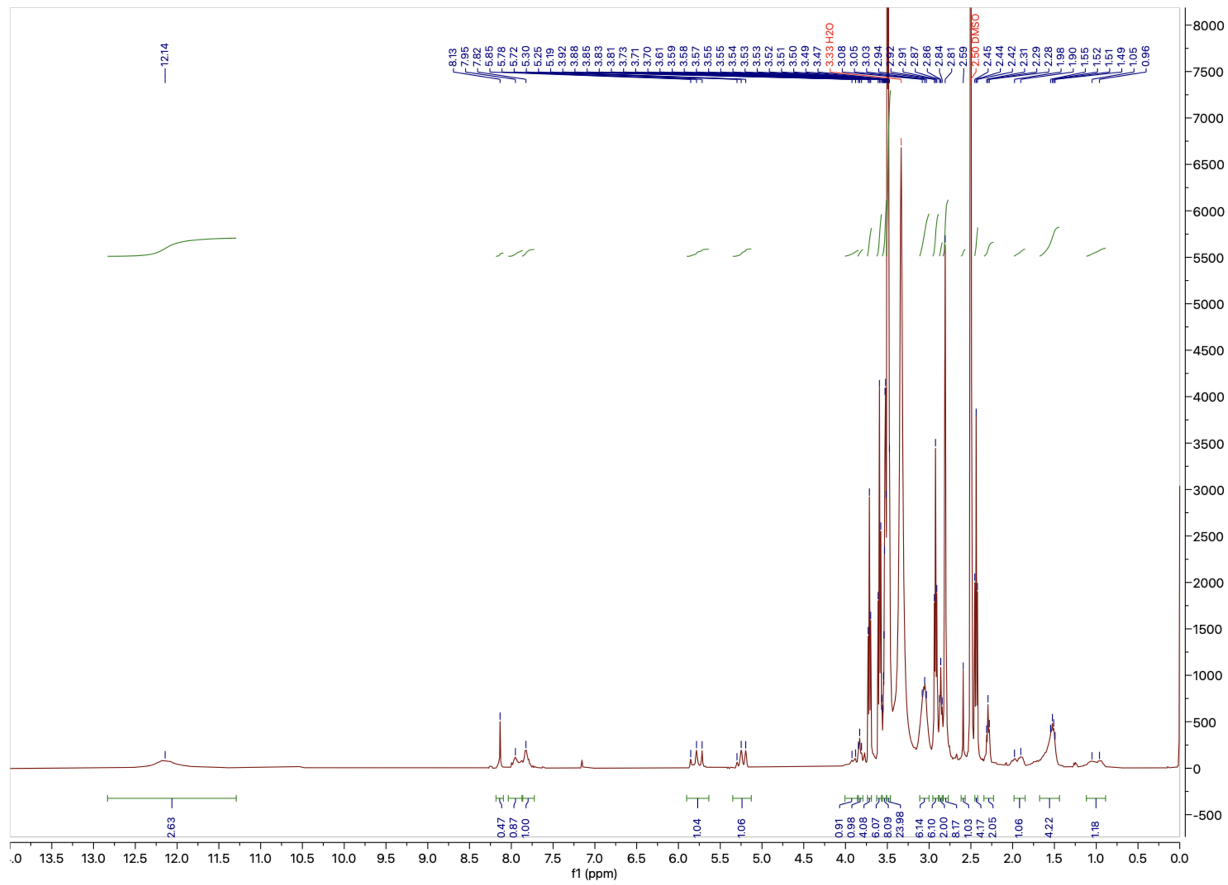

Fig. S4: Proton NMR of DOTA-C_2TCO-NHS

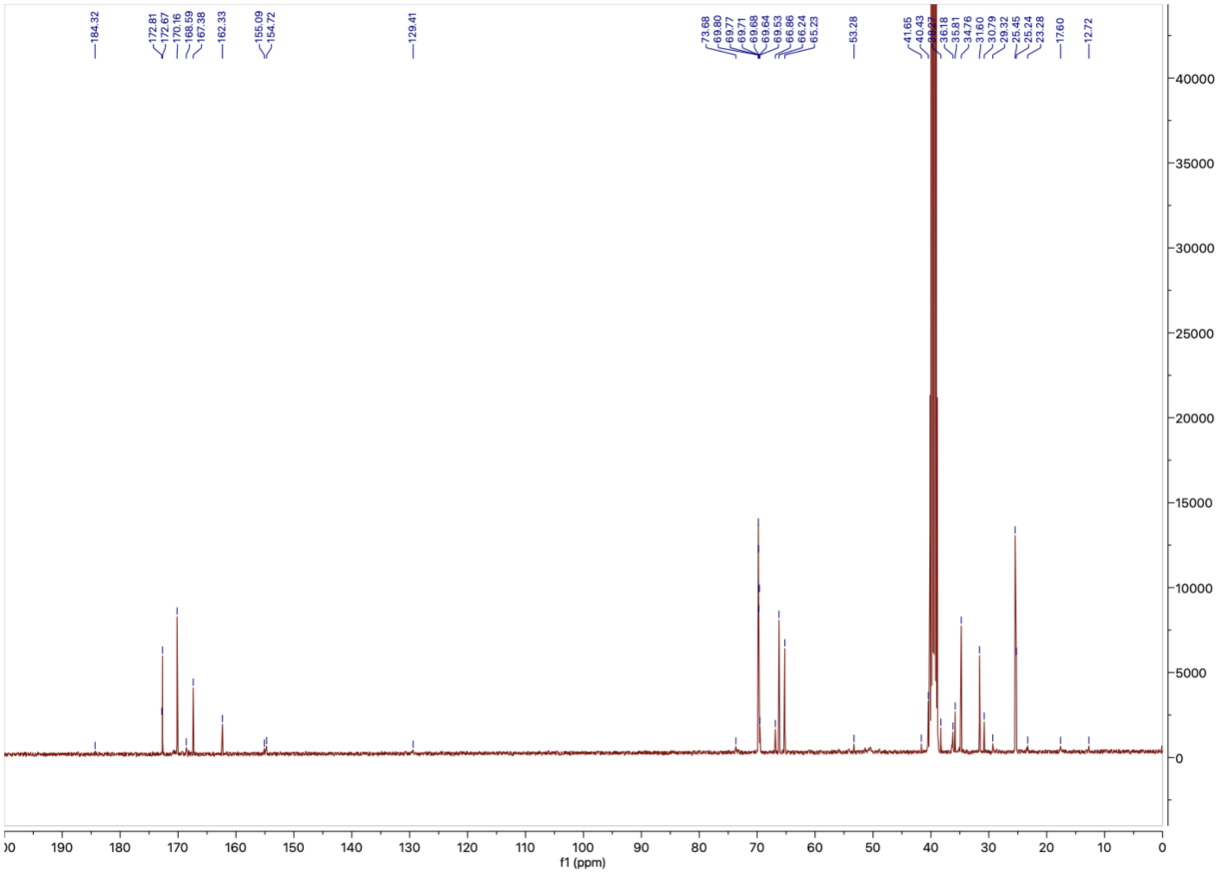

Fig. S5: Carbon NMR of DOTA-C_2TCO-NHS

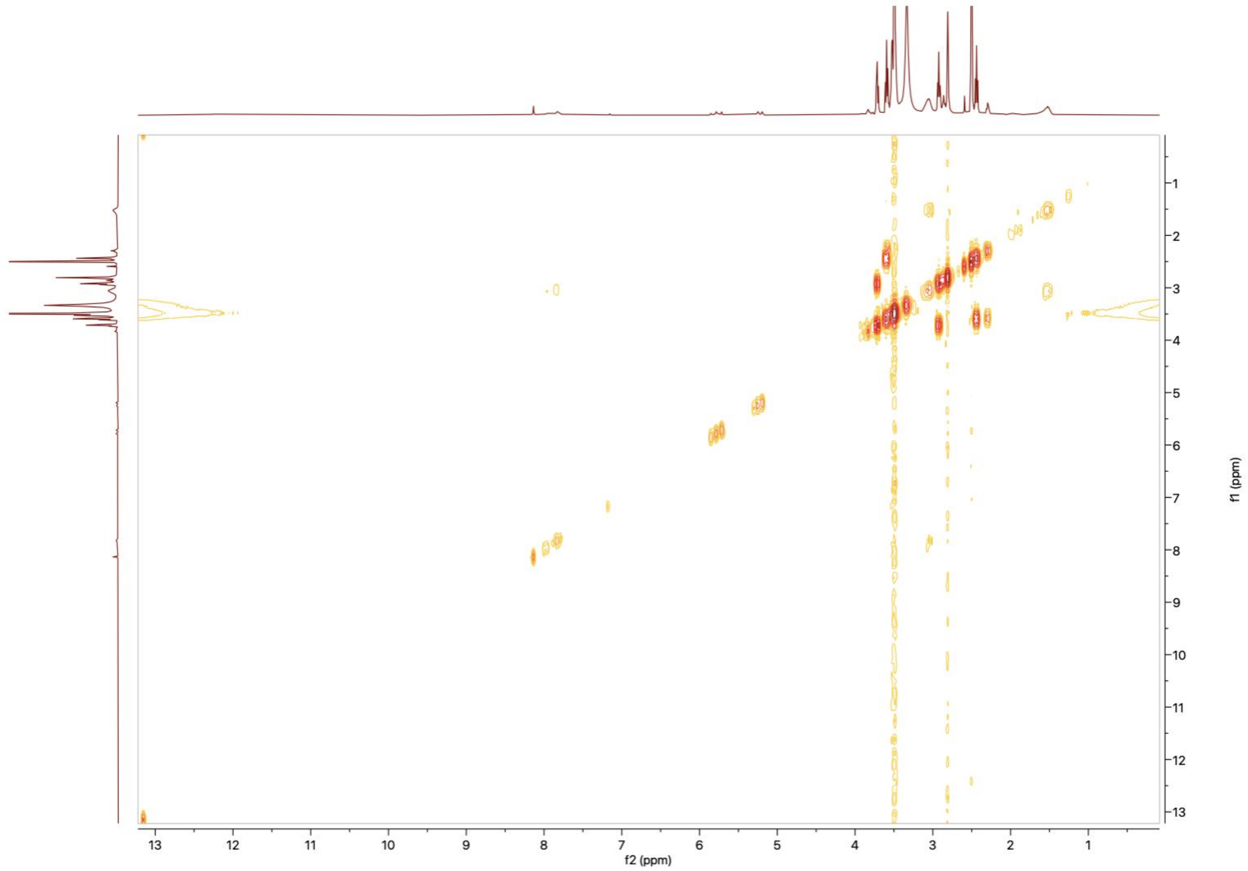

Fig. S6: COSY NMR of DOTA-C_2TCO-NHS

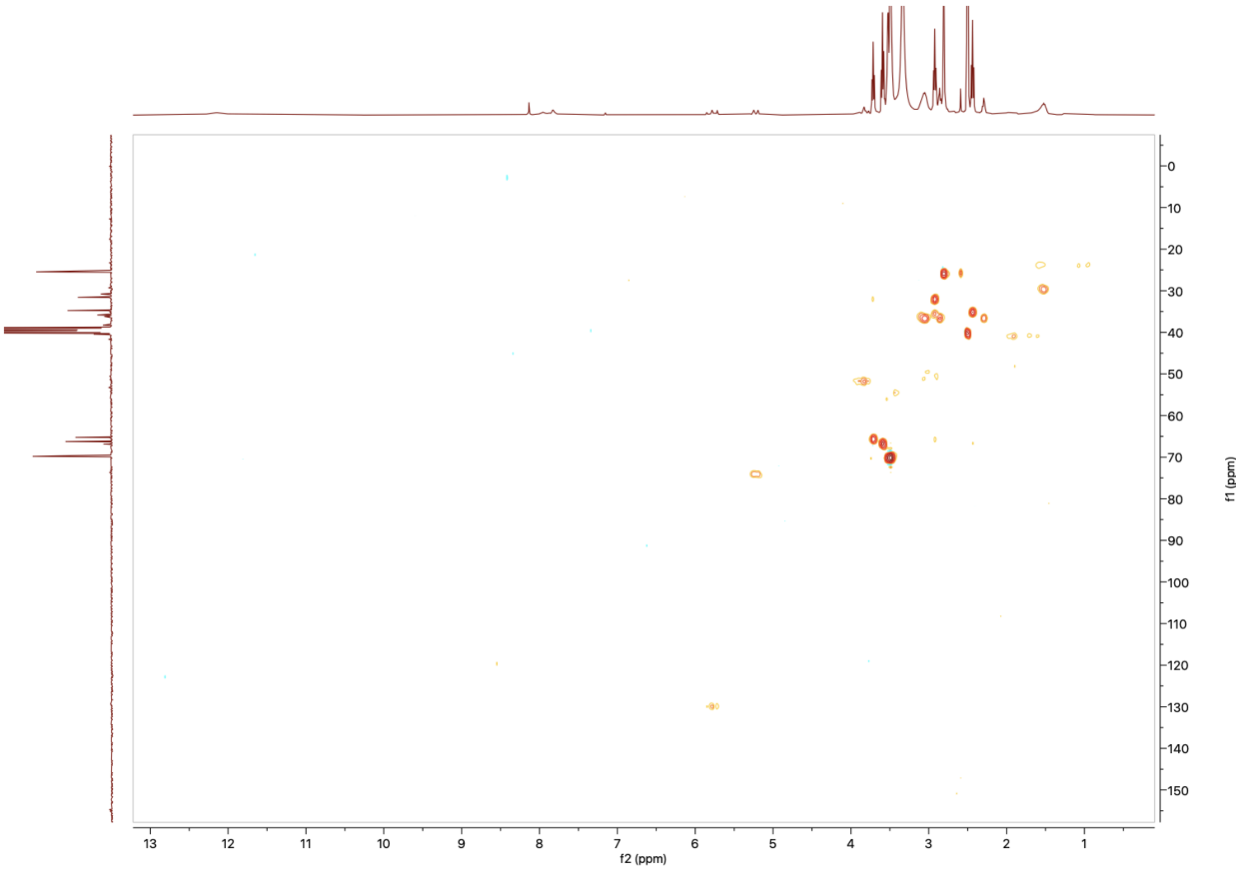

Fig. S7: HSQC NMR of DOTA-C_2TCO-NHS

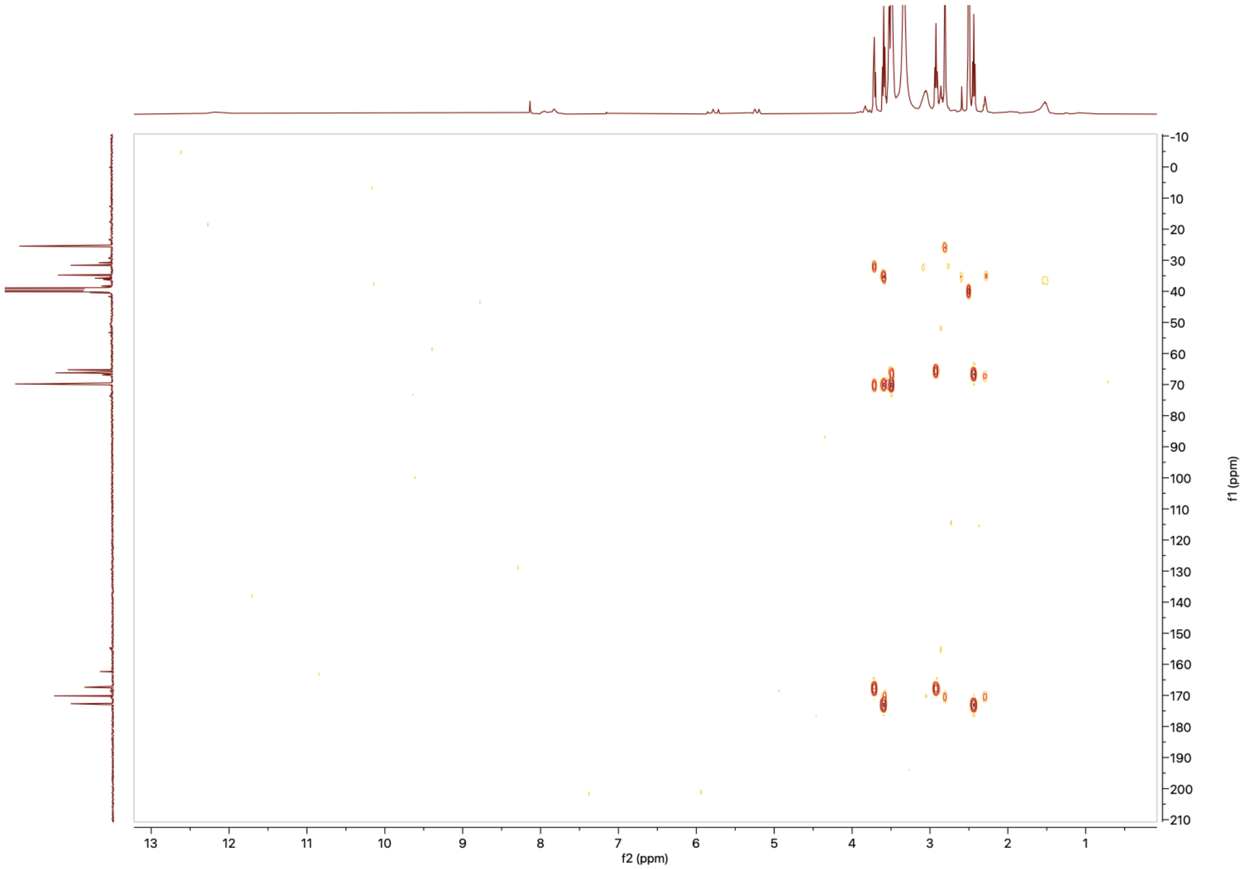

Fig. S8: HMBC NMR of DOTA-C_2TCO-NHS

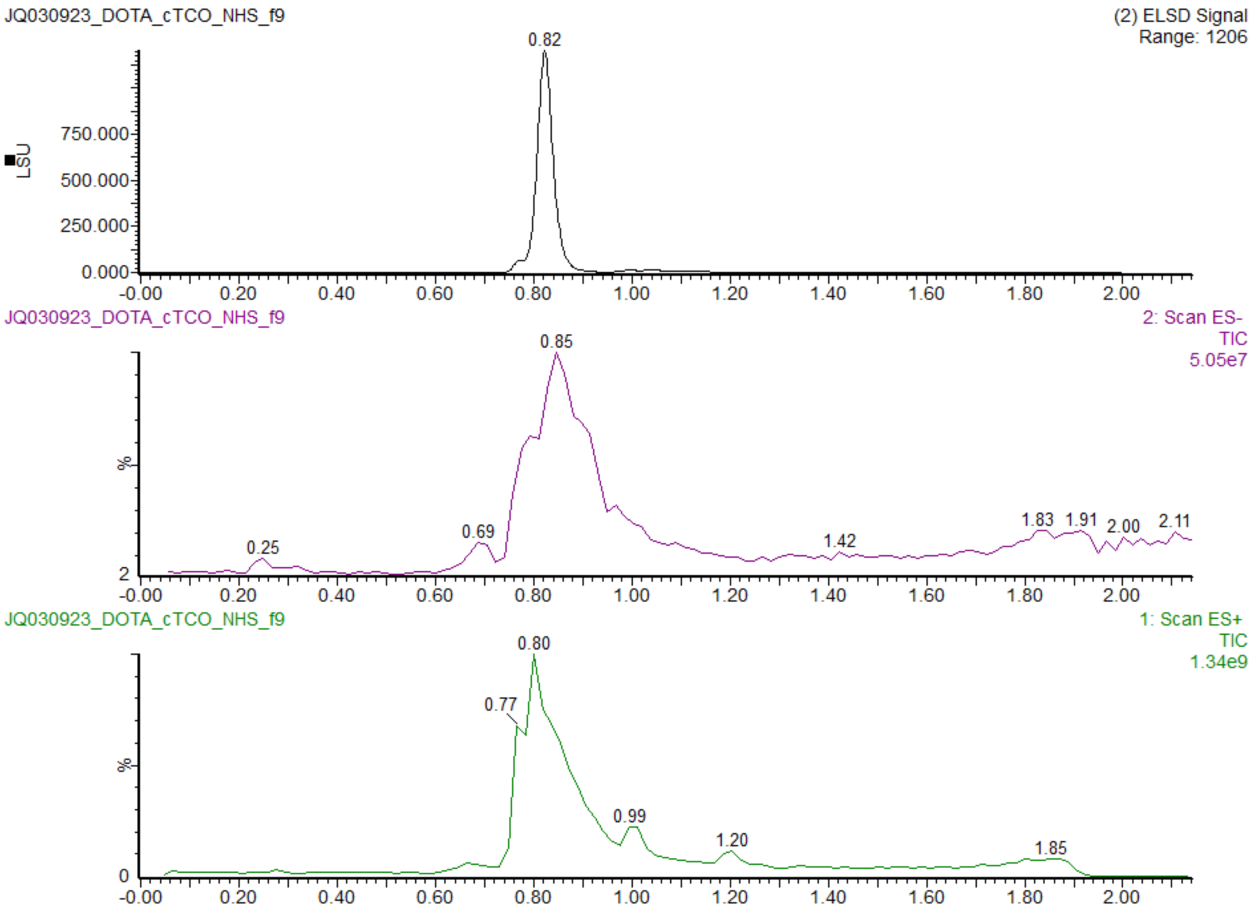

Fig. S9: LCMS chromatographs of DOTA-C_2TCO-NHS

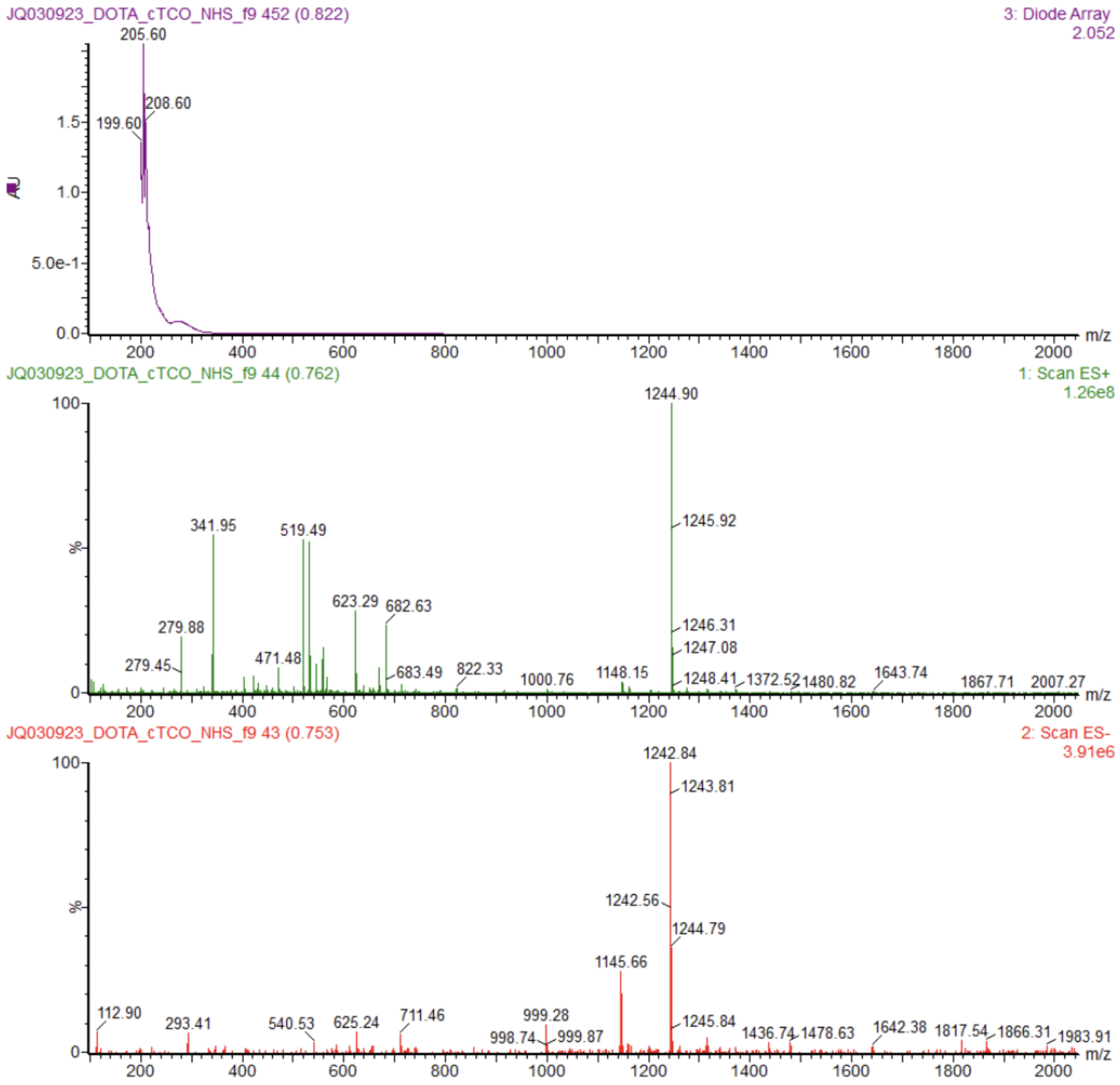

Fig. S10: Spectra of DOTA-C_2TCO-NHS

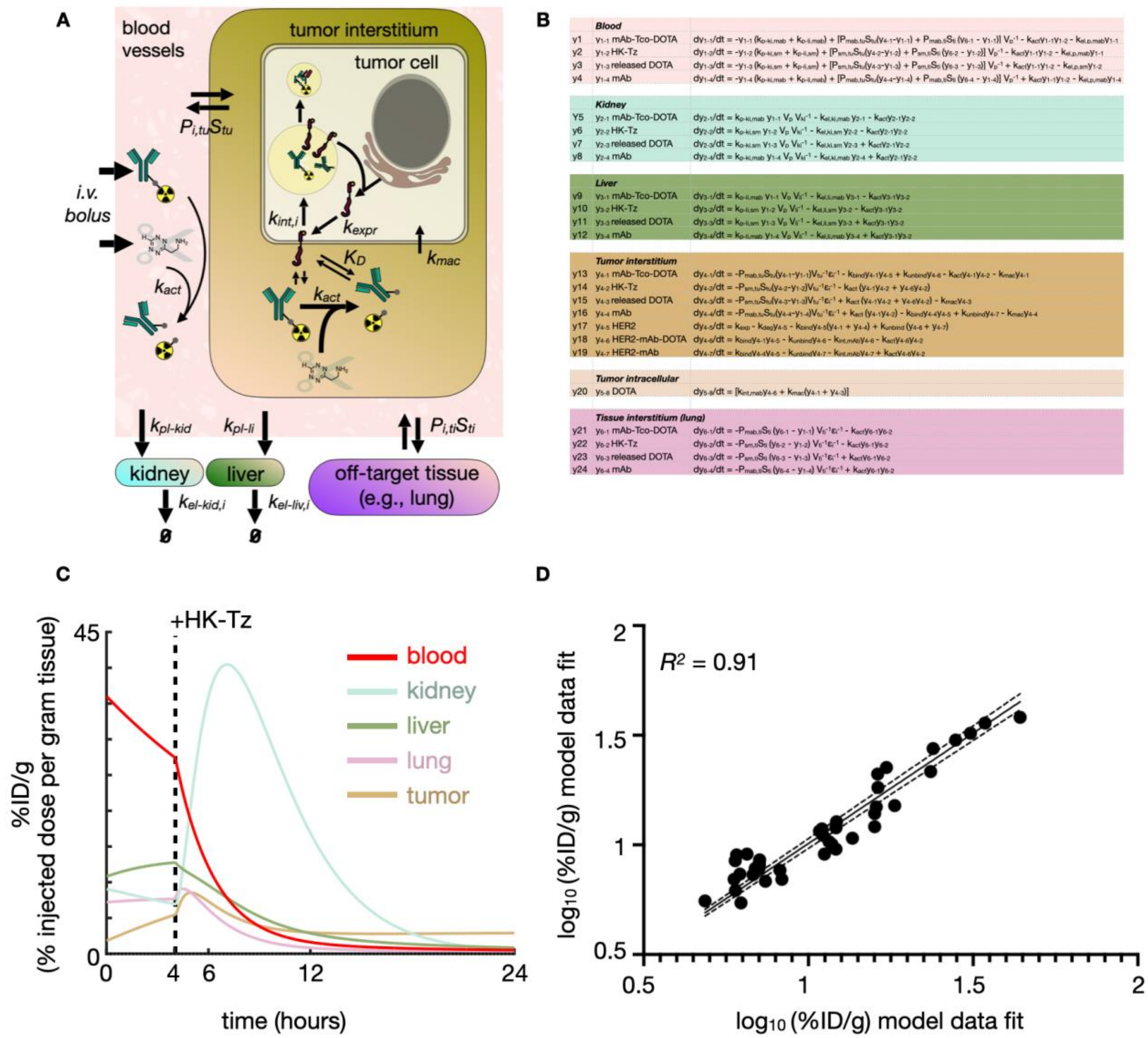

Fig. S11. Model equations and data fit. The computational model of *in vivo* SEMI behavior is shown by a schematic **A**, matching **Fig. 6**) and a corresponding set of coupled ordinary differential equations (**B**). Rate constants and their descriptions are in **Table S1**. **C.** SEMI simulation using the model and optimized rate constants that were fit to the experimental PET data. Signals describe the decay-corrected biodistribution of the Cu-64. **D** Comparison between SEMI simulation and experimental PET data. Each of n = 40 data points represents an individual tissue described in the model at an individual time-point during the time-lapse PET imaging. Spearman correlation, 95% confidence interval, and R^2 values are shown.

Model parameter	Description	Value	Reference	Comment
$k_{p\text{-}ki,mab}$	Kidney mAb uptake from plasma	3.9×10^{-5} min^{-1}	This study	Fit to PET imaging
$k_{p\text{-}li,mab}$	Liver mAb uptake from plasma	6.9×10^{-4} min^{-1}	This study	Fit to PET imaging
$k_{p\text{-}ki,sm}$	Kidney DOTA/Tz uptake from plasma	0.01 min^{-1}	This study	Fit to PET imaging
$k_{p\text{-}li,sm}$	Liver DOTA/Tz uptake from plasma	0.0025 min^{-1}	This study	Fit to PET imaging
$k_{el\text{-}ki,mab}$	Kidney mAb excretion	0.055 min^{-1}	This study	Fit to PET imaging
$k_{el\text{-}li,mab}$	Liver mAb excretion	0.0017 min^{-1}	This study	Fit to PET imaging
$k_{el\text{-}ki,sm}$	Kidney DOTA/Tz excretion	0.006 min^{-1}	This study	Fit to PET imaging
$k_{el\text{-}li,sm}$	Liver DOTA/Tz excretion	0.0091 min^{-1}	This study	Fit to PET imaging
$P_{mab,tu}$	Eff tumor vessel permeability, mAb	1.4×10^{-7} cm s^{-1}	This study	Fit to PET imaging
S_{tu} / V_{tu}	Tumor vascular surface area	34 cm^{-1}	[1]	Highly tumor-dependent
$P_{mab,ti}$	Eff tissue vessel permeability, mAb	0.13×10^{-7} cm s^{-1}	This study	Fit to PET imaging
S_{ti} / V_{ti}	Tissue vascular surface area	250 cm^{-1}	[1]	Lung as model off-target tissue
V_p	Plasma volume	0.75 mL	JAX	C57BL/6J
V_{ki}	Kidney volume (L+R)	0.25 mL	JAX	C57BL/6J
V_{li}	Liver volume	1 mL	JAX	C57BL/6J
V_{tu}	Tumor volume	0.5 mL	This study	
V_{lung}	Lung volume	0.85 mL	JAX	C57BL/6J
ε_i	Interstitial void fraction	0.25	[2]	
k_{act}	Second-order SENIT reaction rate	10.5 M^{-1} s^{-1}	This study	Fit to PET imaging
k_{mac}	Tumor macropinocytosis rate	0.0001 min^{-1}	[3]	highly tumor-dependent
$P_{sm,tu}$	Eff tumor vessel permeability, DOTA/Tz	104×10^{-7} cm s^{-1}	This study	Fit to PET imaging
$P_{sm,ti}$	Eff tissue vessel permeability, DOTA/Tz	104×10^{-7} cm s^{-1}	This study	Simplified to $P_{sm,tu} = P_{sm,ti}$
k_{bind}	mAb/receptor association rate constant	7.1×10^{5} M^{-1}s^{-1}	[4]	trastuzumab / HER2
k_{unbind}	mAb/receptor dissoc rate constant	3.4×10^{-4} s^{-1}	[4]	trastuzumab / HER2
k_{expr}	Receptor baseline expression	$[\text{Target}]_0 \times k_{deg}$	[5]	Maintains Target_0 steady state
k_{deg}	Receptor baseline degradation	0.0036 min^{-1}	This study	Simplified as $k_{deg} = k_{int}$
k_{int}	mAb-bound receptor internalization	0.0036 min^{-1}	This study	Fit to PET, initialized from [6] for trastuzumab
$t_{HK\text{-}Tz}$	Time of dosing HK-Tz	4 hr	This study	
$[\text{Target}]_0$	On-target receptor expression	1.6 μM	[6]	HER2+++ tumor
$[\text{mAb}]_0$	mAb dose (initial conc in plasma)	0.1 nM kg^{-1}	This study	314 μCi / 0.5 μg
$[\text{Tz}]_0$	HK-Tz dose (initial conc in plasma)	3.5×10^{-8} mol kg^{-1}	This study	
$k_{el\text{-}p,sm}$	Remaining plasma elim rate, DOTA/Tz	3.1×10^{-7} min^{-1}	This study	Fit to PET imaging
$k_{el\text{-}p,mab}$	Remaining plasma elim rate, mAb	1.8×10^{-8} min^{-1}	This study	Fit to plasma $t_{1/2}$ 15.7 hr
C_0/C_{inj}	Fraction injection in blood at t=0	0.54	This study	Fit to PET imaging
HCT	Hematocrit	0.5	JAX C57BL/6J	
Tumor VVF	Vessel volume fraction	0.05	[7]	
Lung VVF	Vessel volume fraction	0.2	[8]	
Kidney VVF	Vessel volume fraction	0.25	[9]	
Liver VVF	Vessel volume fraction	0.3	[10]	
Cu-64 $t_{1/2}$	Radioactivity half life	12.7 hr		
NF	Practical imaging detection term	0.4 μCi / mL	This study	Rough approximate from PET imaging for tumor : tissue imaging ratio

Table S1: Summary of modeling parameters

References

(1) Zhang, L.; Bhatnagar, S.; Deschenes, E.; Thurber, G. M. Mechanistic and quantitative insight into cell surface targeted molecular imaging agent design *Sci. Rep.* **2016**, *6*, 25424.

(2) Thurber, G. M.; Weissleder, R. A systems approach for tumor pharmacokinetics. *PLoS One* **2011**, *6*, e24696.

(3) Ng, T. S. C.; Hu, H.; Kronister, S.; Lee, C.; Li, R.; Gerosa, L.; Stopka, S. A.; Burgenske, D. M.; Khurana, I.; Regan, M. S. et al. Overcoming differential tumor penetration of BRAF inhibitors using computationally guided combination therapy. *Sci. Adv.* **2022**, *8*, eabl6339.

(4) Bostrom, J.; Haber, L.; Koenig, P.; Kelley, R. F.; Fuh, G. High Affinity Antigen Recognition of the Dual Specific Variants of Herceptin Is Entropy-Driven in Spite of Structural Plasticity *PLOS ONE* **2011**, *6*, e17887.

(5) Cilliers, C.; Guo, H.; Liao, J.; Christodolu, N.; Thurber, G. M. Multiscale Modeling of Antibody-Drug Conjugates: Connecting Tissue and Cellular Distribution to Whole Animal Pharmacokinetics and Potential Implications for Efficacy *AAPS J.* **2016**, *18*, 1117-1130.

(6) Singh, A. P.; Shah, D. K. Application of a PK-PD Modeling and Simulation-Based Strategy for Clinical Translation of Antibody-Drug Conjugates: a Case Study with Trastuzumab Emtansine (T-DM1). *AAPS J.* **2017**, *19*, 1054-1070.

(7) Bremer, C.; Mustafa, M.; Bogdanov, A.; Ntziachristos, V.; Petrovsky, A.; Weissleder, R. Steady-State Blood Volume Measurements in Experimental Tumors with Different Angiogenic Burdens—A Study in Mice *Radiology* **2003**, *226*, 214-220.

(8) Emond, E.; Holman, B.; Thielemans, K.; Hutton, B.; Koshino, K.; Iida, H. Comparison of blood volume estimated from dynamic ¹⁸F-FDG-PET and ¹⁵O-CO-PET within porcine lungs *J. Nucl. Med.* **2017**, *58*, 698.

(9) Rasmussen, S. N. Intrarenal red cell and plasma volumes in the non-diuretic rat *Pflügers Archiv* **1973**, *342*, 61-72.

(10) Wayson, M. B.; Leggett, R. W.; Jokisch, D. W.; Lee, C.; Schwarz, B. C.; Godwin, W. J.; Bolch, W. E. Suggested reference values for regional blood volumes in children and adolescents. *Phys. Med. Biol.* **2018**, *63*, 155022.
